# H19 regulation of oestrogen induction of symmetric division is achieved by antagonizing Let‐7c in breast cancer stem‐like cells

**DOI:** 10.1111/cpr.12534

**Published:** 2018-10-18

**Authors:** Meng Wang, Yuan Li, Guo‐Dong Xiao, Xiao‐Qiang Zheng, Ji‐Chang Wang, Chong‐Wen Xu, Sida Qin, Hong Ren, Shou‐Ching Tang, Xin Sun

**Affiliations:** ^1^ Department of Thoracic Surgery and Oncology, The Second Department of Thoracic Surgery, Cancer Center First Affiliated Hospital of Xi’an Jiaotong University Xi’an China; ^2^ School of Humanities and Social Sciences Xi’an Jiaotong University Xi’an China; ^3^ Department of Medical Oncology, Cancer Center First Affiliated Hospital of Xi’an Jiaotong University Xi’an China; ^4^ Department of Vascular and Endovascular Surgery First Affiliated Hospital of Xi'an Jiaotong University Xi’an China; ^5^ Department of Otorhinolaryngology First Affiliated Hospital of Xi’an Jiaotong University Xi’an China; ^6^ Cancer Institute, Clinical and Translational Research University of Mississippi Cancer Institute Jackson Mississippi

**Keywords:** cancer stem cells, division modes, MiRNA sponge, Wnt signalling

## Abstract

**Objectives:**

Breast cancer stem‐like cells (BrCSCs) are the major reason for tumour generation, resistance and recurrence. The turbulence of their self‐renewal ability could help to constrain the stem cell expansion. The way BrCSCs divided was related to their self‐renewal capacity, and the symmetric division contributed to a higher ability. Non‐coding long RNA of H19 was involved in multiple malignant procedures; the role and mechanistic proof of non‐coding long RNA of H19 in controlling the divisions of BrCSCs were barely known.

**Materials and Methods:**

Indicative functions of H19 in preclinical study were analysed by using the TCGA data base. Division manners were defined by using fluorescence staining.

**Results:**

We identified the stimulation of H19 on symmetric division of BrCSCs, which subsequently resulted in self‐renewing increasing. H19 inhibited the Let‐7c availability by acting as its specific molecular sponge, and with Let‐7c inhibition, oestrogen receptor activated Wnt signalling was unconstrained. Similarly, restoring Let‐7c constrained oestrogen receptor activated Wnt factors, which sequentially inhibited the H19 decreasing of Let‐7 bioavailability. Let‐7c is reactivated in vitro where H19 was knockdown, and later inhibited the symmetric division of BrCSCs. Reciprocally, Wnt pathway activation leads to H19 increasing, which in turn decreased Let‐7c bioavailability.

**Conclusions:**

Our results revealed a previously undescribed double negative feedback loop between sponge H19 and targeted Let‐7c through oestrogen activated Wnt signalling that dominated in stem cells’ division.

## 1 INTRODUCTION

Breast cancer has topped the cancer incidence chart for years and has become the second reason for cancer‐related mortality around the world,^1^, ^2^ and recurrence is inevitable for chemotherapy‐resistant breast cancers. Strengthened and powerful therapies still have not been able to elevate quality of life.^3^ American and European medical organizations have emphasized the need for new therapy strategies, to more precisely identify what works in clinical practice, and to generate value‐based cancer treatment (https://www.esmo.org/Guidelines, https://www.asco.org/practice-guidelines). The precise therapy, emphasizing the need for new therapy strategies to better identify which clinical practices would generate value‐based cancer treatment, and which therapies target cancer cell regeneration and recurrence, needs targeted to the elimination of the roots for regeneration and recurrence.^4^, ^5^ Sub‐colonies of breast cancer stem‐like cells (BrCSCs) were blamed for lying dormant for a time^6^ and were considered the greatest obstacle to finding a way to cure cancer.^7^, ^8^ Controlling the number of cancer stem cells will help constrain cancer recurrence.^9^, ^10^

Normal stem cells are more likely to divide asymmetrically, to sustain the stem cells pool, and functionally, to generate one daughter cell to differentiate.^9^, ^11^, ^12^ However, division manners were often irregular in cancer stem cells, which tend to divide symmetrically, with unlimited checkpoint.^13^, ^14^ The uncontrolled symmetric division expands the population of stem cells pool and was associated with carcinogenesis and malignancies by a largely unknown mechanism. The components or suppressive non‐coding genes that could regulate the switch between symmetric cell division (SCD) and asymmetric cell division (ACD) will be valuable when trying to repress the self‐renewal capacity.^15^, ^16^

Long non‐coding RNAs (lncRNAs) are lengthy and functional transcripts and were considered as the prospective biomarkers and potential therapeutic targets in cancer diagnosis and treatment.^17^, ^18^ Being implicated in development and growth control, H19 was originally found to be associated with human genetic disorders and recently was identified as one of the major genes in cancer. H19 encodes a 2.6 kb capped, spliced and polyadenylated non‐coding RNA, which is predominantly cytoplasmic.^19^, ^20^ H19 is actively involved in all stages of tumorigenesis and is expressed in multiple cancer types.^21^ Through inhibiting Let‐7 as a molecular sponge, H19 could sequester and regulate the suppressive Let‐7, functioning as oncogenic non‐coding gene. Regulations of Let‐7/LIN28 axis by altering H19 controlled the fate of CSCs of triple negative breast cancer,^22^ but how H19 functioned in CSCs from Lumina A/B breast cancer was unknown. Considering that majority of breast cancer patients featured the phenotype of Lumina A/B; therefore, finding a way to help avoid long‐term resistance and recurrence is crucial to find a breast cancer cure. Increased H19 allows cancer cells survive through treatment,^21^ and the implicated mechanism of H19 in regulation of BrCSCs’ division will reveal prospective approaches to constrain stem cells invasion.

## 2 MATERIALS AND METHODS

### 2.1 Cell culturing, mimics/inhibitors transfection and lentiviral transduction

Breast cancer cell lines of MCF‐7, ZR75‐1 and HEK‐293T were purchased from American Type Culture Collection and were maintained in DMEM (Gibco, Rochester, NY, USA) or in RPMI 1640 Medium (Hyclone, Logan, UT, USA), respectively. About 10% FBS (Gibco), 1% penicillin and 1% streptomycin (Hyclone) were mixed into medium. For constructing cells with inhibited H19, cells were transfected with specific anti‐H19 inhibitor vector, by using Lipofectamine 3000 as directed in the manufacturer's instruction, and stable cell lines were selected with neomycin (800 μg/mL).

### 2.2 Quantitative real‐time PCR and western blot

Total mRNA was reverse‐transcribed into cDNA (AT301 TransGen Biotech, Beijing, China), and real‐time quantitative PCR was performed with CFX96 Real‐Time PCR Detection System (Bio‐Rid, Hercules, CA, USA). For western blot, the protein from cell extracts was separated by 10% SDS‐PAGE electrophoresis and was later transferred onto PVDF membrane. Membranes were incubated with ESR1 (1:3000, EPR4097, ab108398; Santa Cruz, Dallas, TX, USA), TCF4 (1:2000, ab217668; Abcam, Cambridge, MA, USA), CMYC (1:2000, ab32072; Abcam), LEF1 (1:2000, ab137872; Abcam), WNT1 (1:1000, ab15251; Abcam), CTNNB1 (1:1000, C2206; Sigma‐Aldrich, St. Louis, MO, USA), VINCULIN (1:5000, #18799; Cell Signaling Technology, Danvers, MA, USA), and then detected using ECL Blotting Detection Reagents (Merck Millipore, Burlington, MA, USA). Cells of different groups were suspended in DMEM/F12 Medium supplemented with 20 ng/mL EGF (BD Biosciences, San Jose, CA, USA), bFGF and 4 μg/mL insulin (Sigma), and then plated in 6‐well ultra‐low attachment dishes (1000 cells/mL; Corning Incorporated, Tewksbury, MA, USA). To analyse the self‐renewal ability, sphere number of each captured image was counted by using phase contrast microscope (Nikon, Tokyo, Japan).

### 2.3 Immunofluorescence and EdU staining

Cells were blocked with bovine serum albumin (BSA) at 37°C for 30 minutes and then were incubated with CTNNB1 antibody (1:200, Rabbit monoclonal, C2206; Sigma‐Aldrich) at 4°C overnight, followed by Goat Anti‐Rabbit IgG (Alexa Fluor® 568, 1:1000; Abcam) for 1 hour at room temperature. The nuclei were counterstained with 4, 6‐diamidino‐2‐phenylindole (DAPI, 1:10000; 4084; Cell Signaling). For EdU labelling assay, cells isolated from spheres were treated with trypsin and accutase, and were seeded into chamber slides after being filtered into singled cell (Nunc; Thermo Scientific, Carlsbad, CA, USA; Figure S1A). EdU intensity was detected after cells were incubated with 10uM EdU for staining 24 hours. Detection was performed using Click‐iT Plus EdU Alexa imaging Kit (MP 10637; Life Technologies, Carlsbad, CA, USA). The fluorescence images were obtained using an Olympus microscope, and the counting procedure was performed on twenty images that captured from each single experiment (Figure S1B).

### 2.4 Luciferase reporter assays

Grouped cells of MCF‐7, ZR75‐1 and HEK293 were seeded into 24‐well plates (2 × 10^5^ per well) before transfection. Pre‐treated cells were co‐transfected with either 300 ng luciferase reporter vectors (H19‐3′UTR‐wt or H19‐3′UTR‐mt) or 30 nmol/L Let‐7c mimics. Renilla luciferase plasmid (100 ng/well; Promega, Madison, WI, USA) was used as an internal control. The promoter plasmid of wild‐type pGL3‐LEF/TCF reporter (wt‐TCF4, TOP) or pGL3‐LEF/TCF reporter (mut‐TCF4, FOP) was co‐transfected with certain genes or agents. For detection of HEK‐293T cells, cells were co‐transfected with 200 ng TOP or FOP plasmid, 25 nmol/L Let‐7c mimics or specific agents, 20 ng pRL‐TK Renilla luciferase vector (Promega) as normalization vector. Forty‐eight hours after transfection, cells were harvested and lysed for standard luciferase assay. Luciferase activities (Firefly and Renilla) were measured using the Dual‐Luciferase Reporter Assay System (Promega) and normalized to Renilla luciferase activity.

### 2.5 Statistical analysis

Numerical data were presented as mean ± standard deviation (SD), of three‐six independent experiments. Statistical analysis was carried out by using Graph Pad Prism 6 (La Jolla, CA, USA) and Microsoft Excel software (Redmond, WA, USA). Differences between groups were assessed by using Student's *t* test or ANOVA individually. *P* < 0.05 was statistically significant.

## 3 RESULTS

### 3.1 Let‐7 family of miRNAs were associated with better prognosis

**Figure 1:**
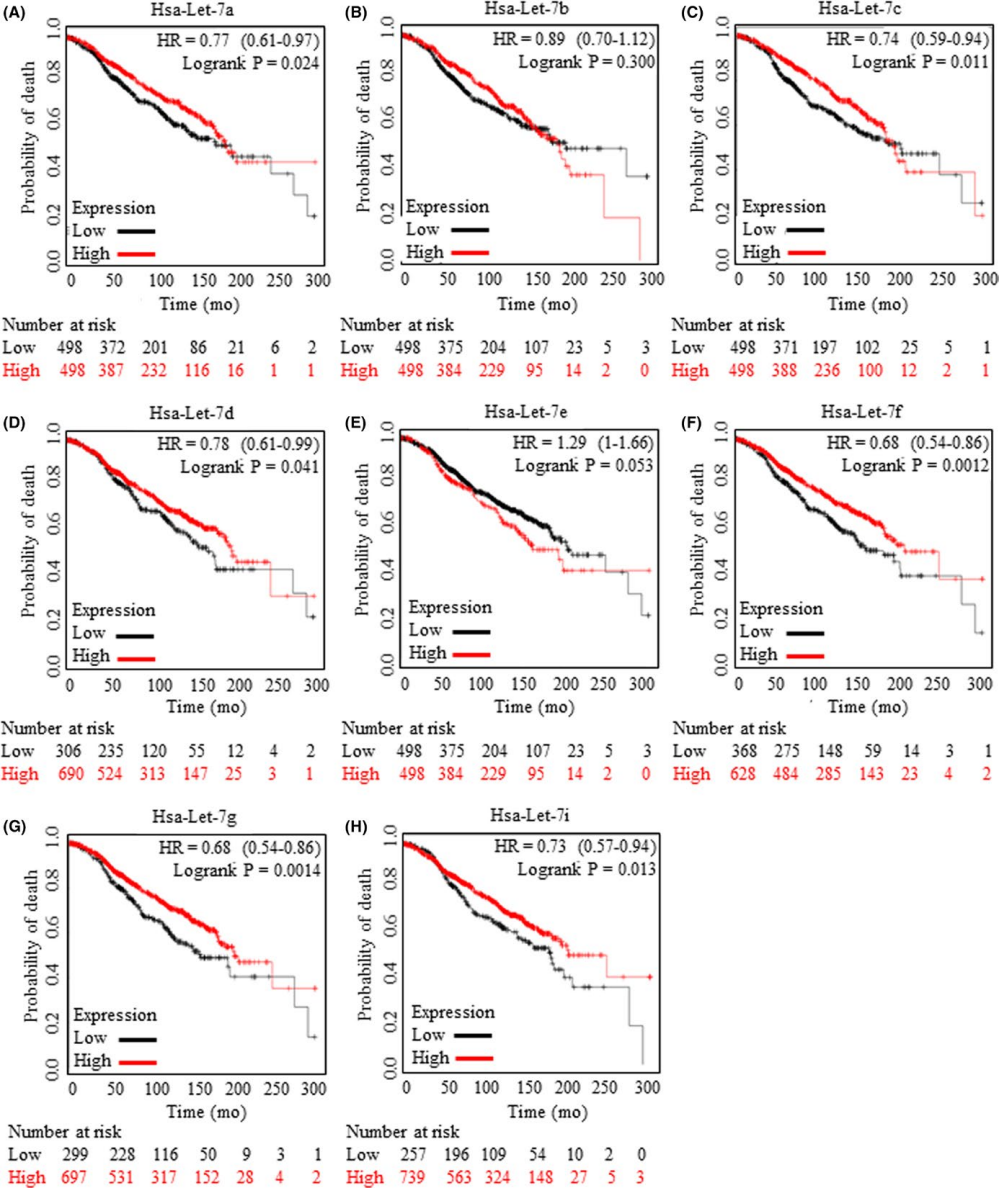
Increased expression levels of Let‐7 miRNAs were referred to better prognosis. Kaplan‐Meier analysis (https://kmplot.com/analysis/) was carried out to explore the indicative roles of Let‐7 miRNAs in predicating clinical outcomes. Let‐7 family members were all subjected for survival analysis, and results of Let‐7a (A), Let‐7b (B), Let‐7c (C), Let‐7d (D), Let‐7e (E), Let‐7f (F), Let‐7g (G) and Let‐7i (H) were generated and cited. Increased Let‐7 family members of Let‐7a, Let‐7c, Let‐7d, Let‐7f, Let‐7g and Let‐7i were related to longer survival time

Correlation between Let‐7 family of miRNAs and prognosis of patients with oestrogen receptor‐positive breast cancer was studied with applying Kaplan‐Meier analysis (https://kmplot.com/analysis/).^23^, ^24^, ^25^ Results of Let‐7a (Figure 1A), Let‐7b (Figure 1B), Let‐7c (Figure 1C), Let‐7d (Figure 1D), Let‐7e (Figure 1E), Let‐7f (Figure 1F), Let‐7 g (Figure 1G) and Let‐7i (Figure 1H) were generated and captured. Let‐7c, whose overexpression was proved to be positively correlated with Wnt signalling activity inhibition,^7^ was identified as one protective indicator when predicating prognosis.

### 3.2 Wnt signalling was hyperactivated in breast cancer tissues

**Figure 2:**
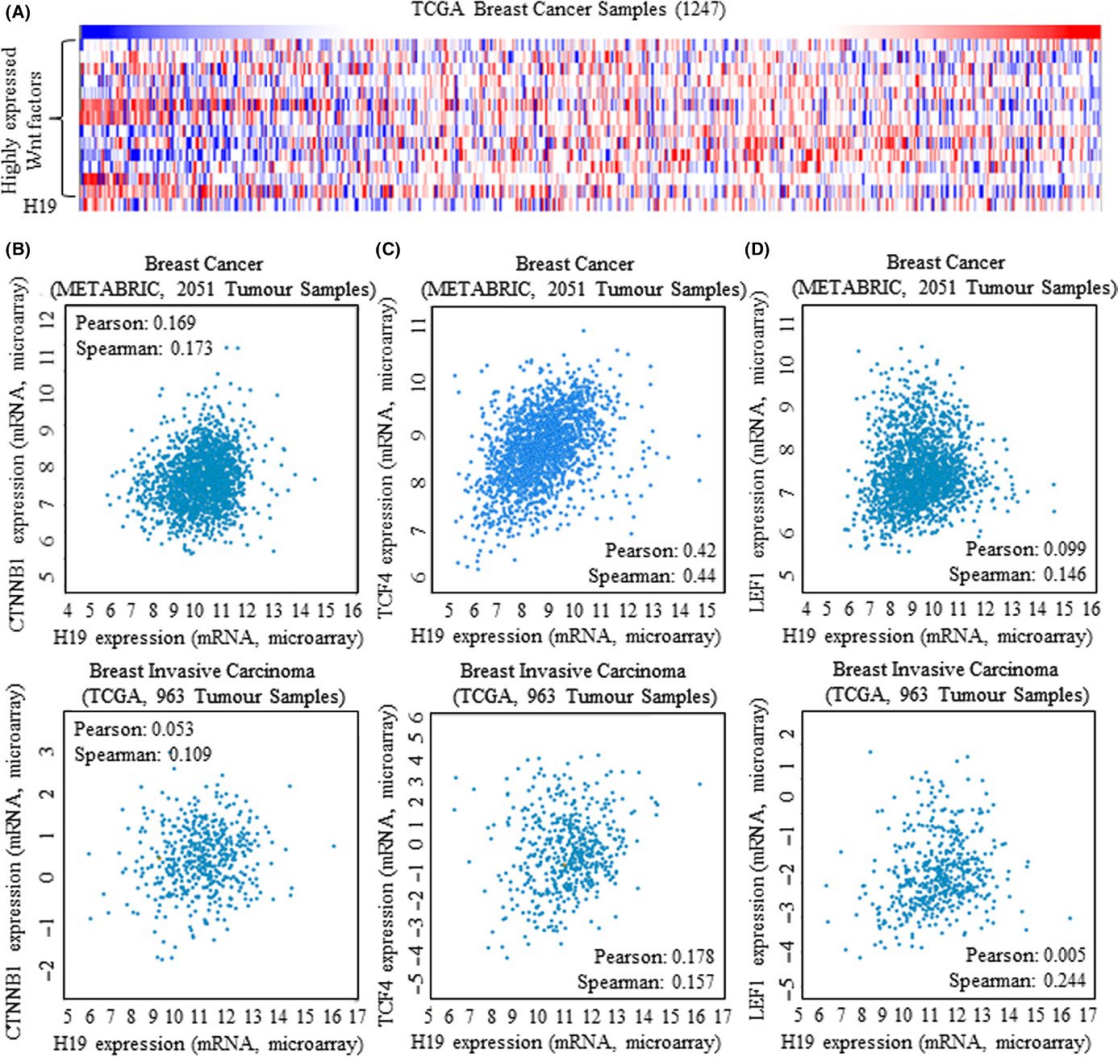
Wnt activation and increased H19 in breast cancer tissues. Data base of online TCGA and METABRIC was applied for mining information of signalling functions. A, Heatmap result of Wnt signalling factors and H19, and genes of “APC APC2 BBC3 FBXW11 SMAD1 SMAD2 SMAD3 SMAD4 SOX2 ACTG1 CHD1 CTNNB1 CTNNA1 CTNNA2 CTNNA3 CCND1 CCND2 CCND3 DVL1 DVL2 DVL3 FZD1 FZD2 FZD3 FZD4 FZD5 FZD6 FZD7 FZD8 FZD9 FZD10 LEF1 MPP5 NF2 SNAI1 SNAI2 TCF7 TCF7L1 TCF7L2 TGFB1 TGFB2 TGFB3 TGFBR1 TGFBR2 MYC” were arranged in order from the top down, with H19 being at the bottom. B, Data of TCF4 were acquired from both METABRIC of 2051 tumour samples (above), and TCGA of 963 tumour samples (below). C, Data of LEF1 were acquired from both METABRIC of 2051 tumour samples (above), and TCGA of 963 tumour samples (below). The correlation was calculated and labelled as Pearson and Spearman score in figure

Wnt signalling factors were analysed for their potential correlation with overall survival (OS) and recurrence‐free survival (RFS) by applying Kaplan‐Meier analysis (https://kmplot.com/analysis/).^23^, ^24^, ^25^ RNA sequence result showed the universally overexpressed Wnt signalling factors in tissues of breast cancer (Figure S2A), and Heatmap result of Wnt pathway further revealed the activation of Wnt signalling in breast cancer (Figure 2A, Figure S2B). Irregular upregulation of representative Wnt signalling factors of CCND1 (Figure S3A), SOX2 (Figure S3B), MAPK1 (Figure S3C) and CMYC (Figure S3D) was correlated to either decreased free survival or shorter disease‐free survival.

### 3.3 Correlation between Wnt signalling and H19 expression

The long non‐coding H19 correlates with higher risk of cancer death in multiple cancers,^22^, ^26^ and Heatmap result of TCGA data base revealed the co‐overexpression of both H19 and Wnt factors (Figure 2A, Figure S2B). The validated data from CBIOPORTAL FOR CANCER GENOMIC (https://www.cbioportal.org/)^27^, ^28^ further revealed the positive correlation between CTNNB1 and H19 (Figure 2B), TCF4 and H19 (Figure 2C), LEF1 and H19 (Figure 2D), from open data of METABRIC and TCGA data base, respectively.

### 3.4 Axitinib inhibition of Wnt activity and Let‐7c overexpression increased the asymmetric cell division in BrCSCs

**Figure 3:**
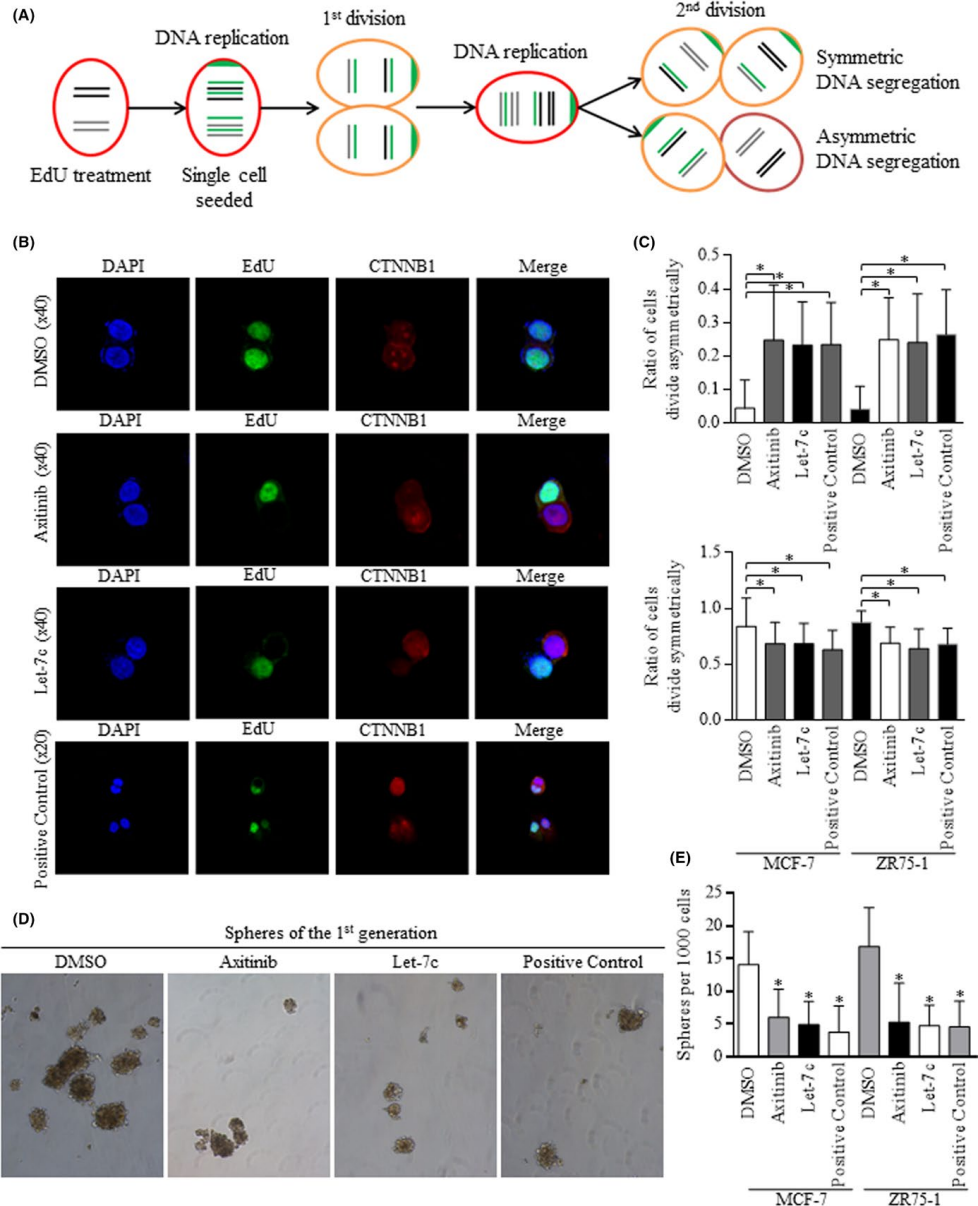
Enforced Let‐7c and Axitinib addition both stimulated the asymmetric division of stem cells. A, Illustration of the EdU dye labelled DNA separation was drafted to help defining the division manners of breast cancer stem cells. B, EdU dye was set up with 488 of green light, and the 568 of red light was also stained to show the asymmetric distribution of CTNNB1. C, Let‐7c overexpression and Axitinib addition stimulated the asymmetric division (above) and decreased the symmetric division (below), comparing to control group, and Nutlin‐3 treatment was set as the positive control. *P < 0.01. D‐E, In spheres formation assay, Let‐7c overexpression and Axitinib addition greatly decreased the number of spheres in the 1st generation, as Nutlin‐3 (positive control) did

A subgroup of CSCs tended to divide symmetrically, generating two unique daughter cells, which will inherit full characteristics of mother cells, especially highlighted for their strong ability to self‐renew. Asymmetric division generated two daughter cells with diverse natures, one of which obtained the strong self‐renewal ability of mother cell, while another one differentiated into certain phenotypes. EdU label‐release assay was applied to define the division modes,^11^, ^13^ which could be segregated into daughter cells, as was illustrated in Figure 3A. Division manners could be defined by EdU staining distribution, similar to previous report (Figure 3B). Both Axitinib and Let‐7c induced cells to divide asymmetrically (above, Figure 3C); however, the ratio of symmetric division was inhibited when treating cells with Axitinib or transfecting cells with Let‐7c (below, Figure 3C). Alternations of division manners resulted in different spheres forming efficiency, and both Axitinib and Let‐7c inhibited the self‐renewal ability of BrCSCs (Figure 3D,E). Groups treated with 10 uM of Nutlin‐3, the MDM2 (mouse double minute 2) antagonist as p53 pathway activator, were set as the positive group, which could stimulate the asymmetric division.

### 3.5 H19 stimulated the symmetric division of BrCSCs

**Figure 4:**
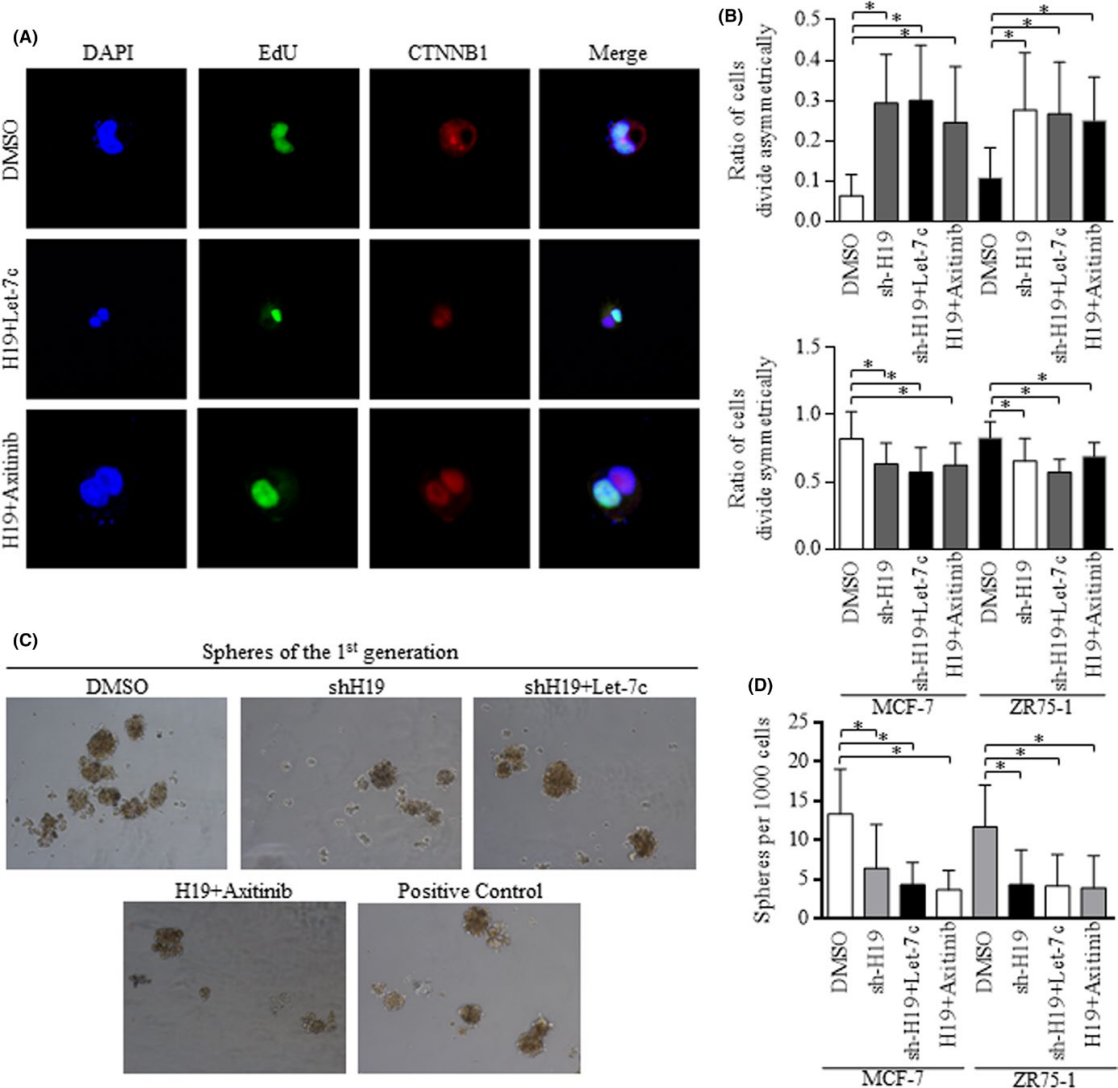
H19 promotes the self‐renewal and symmetric division of cancer stem cells probably in a Let‐7c/Wnt signalling way. A,B, H19 inhibition promotes the asymmetric division of breast cancer stem cells, defined by EdU dye staining, and no significant differences were detected when comparing the groups of shH19 and groups of H19 inhibition with addition of either Let‐7c or Axitinib, suggesting the possible H19/Let‐7c/Wnt signalling. C,D, Groups of H19 inhibition and groups of H19 inhibition with addition of either Let‐7c or Axitinib all decreased the number of spheres, suppressing the self‐renewal ability

H19 inhibition was achieved by lentiviral infection, and the efficiency was about 70% by applying (qRT‐PCR), which increased the asymmetric division ratio greatly, and Axitinib attenuated the H19 stimulation of symmetric division (Figure 4A,B). As was speculated, no significant difference occurred between groups of shH19 and groups of H19 inhibition with Let‐7c overexpression (Figure 4B). Through applying spheres forming assay, we found the H19 inhibition decreased the sphere number significantly, and in groups with enforced H19, Axitinib abolished the H19 stimulation of self‐renewal (Figure 4C,D). Given that Let‐7c suppressed Wnt signalling cascade in CSCs, and increasing Let‐7c did not further strengthen the ACD in groups of H19 inhibition, we hypothesized the functional signalling cascade of H19/Let‐7c/Wnt pathway may exist.

### 3.6 H19 stimulation of symmetric division was dependent on Let‐7 mediation of ESR1 functional pathway

**Figure 5:**
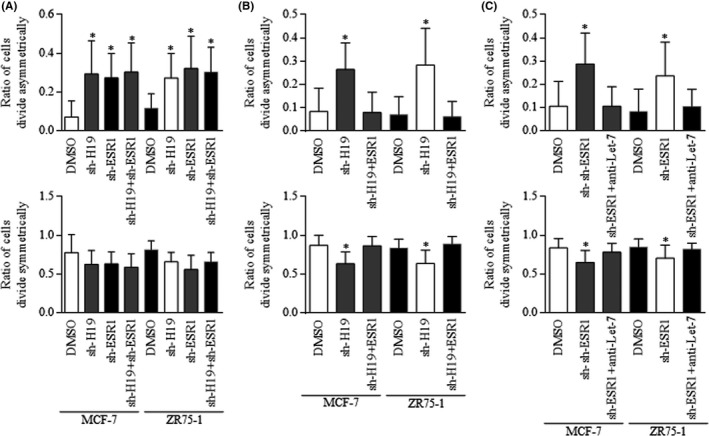
H19 regulation of Let‐7c and ESR1 signalling. A, H19 inhibition and ESR1 inhibition increased the ACD of BrCSCs (above) and decrease the SCD significantly (below). But the combined H19 inhibition and ESR1 inhibition did not further manipulate the division manners. B, Restoring ESR1 abolished the effect of H19 inhibition when activating the ACD (above), inhibits SCD of BrCSCs (below). C, Let‐7c inhibition abolished the ESR1 inhibition induced ACD (above), reactivated SCD of BrCSCs (below)

To study the functional roles of oestrogen receptor‐related pathways, cells were cultured using medium with charcoal‐serum, and with oestrogen as basic supplement nutrient. Either H19 inhibition or ESR1 inhibition increased the ACD of BrCSCs (Figure 5A, above) and decreased the SCD significantly (Figure 5A, below). However, the combination of H19 inhibition and ESR1 inhibition did not further stimulate the ACD (Figure 5A). Introducing ESR1 into cells harbouring H19 inhibition reactivates ACD (Figure 5B), indicating that H19 functioned through ESR1 existence. Inhibiting Let‐7c in cells harbouring ESR1 suppression decreased the ratio of ACD (Figure 5C, above), reactivated SCD of BrCSCs (Figure 5C, below), which strongly suggested that Let‐7c inhibition is critical for H19 functions, and could be mediated by the existence of ESR1.

### 3.7 The existence of ESR1 was critical for H19 induction of Wnt signalling action

**Figure 6:**
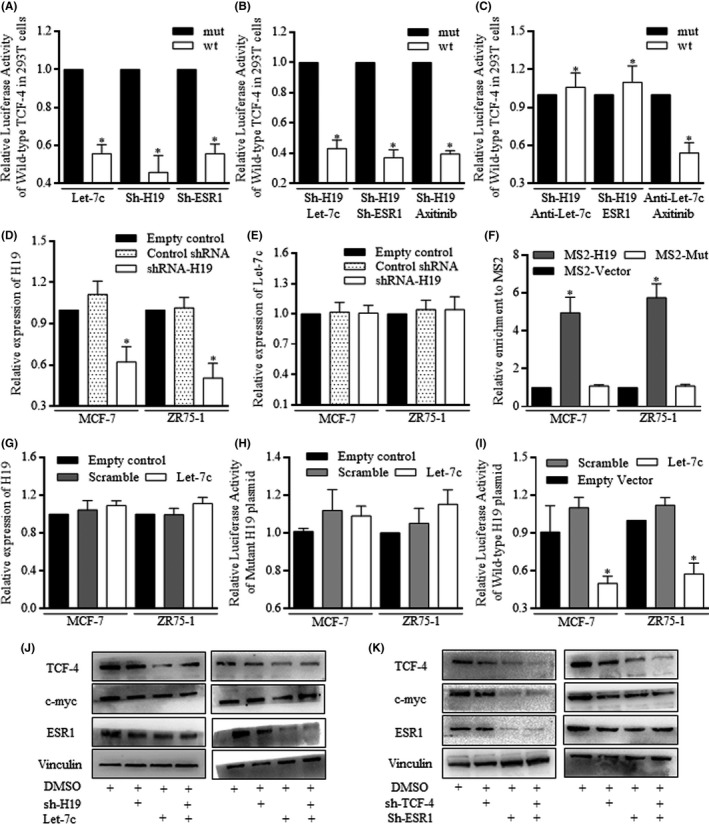
H19 induction of Wnt signalling action. A, Increasing Let‐7c, decreasing H19 and ESR1 all decreased the TCF‐4 promoter activity. B, Decreasing H19 in 293T cells of Let‐7c increasing, ESR1 inhibiting, or Axitinib addition made no significant difference in three groups. C, Inhibiting Let‐7c and restoring ESR1 greatly abolished the H19 inhibition suppressed Wnt activity. In groups treated with Axitinib, Let‐7c inhibition failed to reactivate TCF‐4 promoter. D,E, H19 inhibition did not consequently restore Let‐7c level, failing to degrade Let‐7c expression after connection. F, The Let‐7c‐specific binding site towards to H19 MS2 was effectively grouped with H19. G, Let‐7c did not affect the H19 expression. H,I, Let‐7c degraded the H19 binding site of wild‐type mRNA effectively and failed in groups of mutant mRNA. J,K, Western blotting revealed the H19 controlling of Let‐7c contributed to ESR1 and Wnt activity deregulation, and ESR1 mainly functioned through Wnt signalling

Increasing Let‐7c, decreasing H19 and ESR1, all contributed to TCF‐4 promoter activity inhibition (Figure 6A). When sh‐H19 vector was used in combination with Let‐7c, ESR1 inhibition or Axitinib, no significant difference was detected among these three groups (Figure 6B), suggesting the similar roles of Let‐7c, ESR1 and Wnt signalling as downstream effectors of H19. Both Let‐7c inhibition and ESR1 restoration abolished the role of H19 inhibition, and in groups treated with Axitinib, Let‐7c inhibition failed to reactivate TCF‐4 promoter (Figure 6C).

To identify the functional regulation of H19 on Let‐7c, we constructed the MS2‐associated H19‐Let‐7c binding site to perform the RNA immunoprecipitation, which could pull down H19 and MS2, referring to Let‐7c binding site. H19 inhibition did not restore Let‐7c level and failed to degrade Let‐7c expression after connection (Figure 6D,E). The binding site between H19 and Let‐7c was proven as the binding site‐specific MS2, which was effectively grouped with H19 (Figure 6F). Alternatively, Let‐7c exerted no significant repression on H19 expression, likely proving the inactive combination (Figure 6G).

Enforced Let‐7c degraded the H19 binding site of wild‐type effectively, proving the mechanistic regulation of a competitive sponge molecular, and the combination site has dual characteristics (Figure 6H,I). Western blotting was further used to confirm the mechanistic functions of H19 mediation of Wnt signalling, and H19 controlling of Let‐7c contributed to ESR1 and Wnt activity deregulation (Figure 6J), and ESR1 mainly functioned through Wnt signalling (Figure 6J).

### 3.8 Regulatory loop of H19/Let‐7c/ESR1/Wnt circle

**Figure 7:**
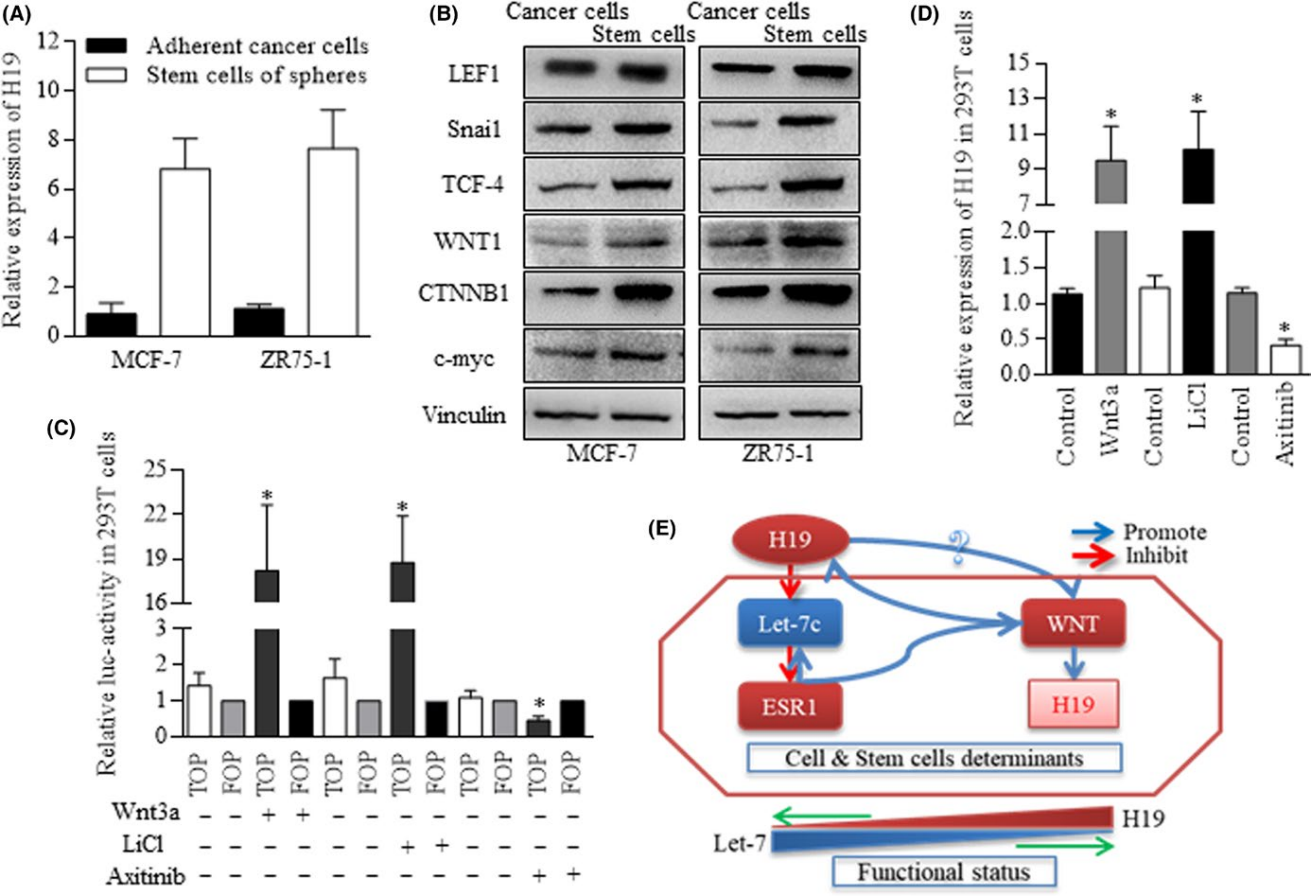
Circular loop of H19/Let‐7c/ESR1/Wnt cascade. A, H19 increased significantly in spheres of BrCSCs. B, Key factors of Wnt signalling increased in the stem cells of MCF‐7 and ZR75‐1 cells. C, Agents of 5 mmol/L LiCl or 400 ng/mL of recombinant mouse Wnt3a protein (Wnt3a) induced the TCF‐4 promoter activity, D, and effectively stimulated the H19 expression level. E, The key function of H19 in manipulation of stem cells’ division manners was dependent on the Wnt signalling activation, and Let‐7c manipulation of ESR1 was critical for connecting the H19 and Wnt in the regulatory loop

Wnt signalling was naturally activated in malignancies and was more greatly in cancer stem‐like cells. We confirmed the overexpressed H19 in spheres of BrCSCs (Figure 7A), and the similar results of Wnt factors were also found in the stem cells of MCF‐7 and ZR75‐1 cells (Figure 7B). To further identify the putative effect of Wnt on H19 expression level by using either 5 mmol/L LiCl or 400 ng/mL of recombinant mouse Wnt3a protein (Wnt3a), both of which induced the TCF‐4 promoter activity significantly (Figure 7C), and later effectively stimulated the H19 expressing. Similarly, blocking Wnt cascade decreased the H19 expression (Figure 7D). The key function of H19 was dependent on the Wnt signalling activation, and Let‐7c manipulation of ESR1 was critical for connecting the H19/Wnt regulatory loop (Figure 7E).

## 4 DISCUSSION

Long non‐coding RNA regulated miRNAs have attracted much attention since the interactions between non‐coding genes paved the way of viewing a whole new mechanistic micro‐world of cancer cell biology.^29^ Cancer stem cells stand steady and are viewed as the obstacle in the way of curing breast cancer, and up to now, no effective and applicable agents or methods have been carried out in treating patients with breast cancer. The progress of inventing anti‐cancer agents and molecules has continuously brought hope of a bright future; however, recurrence and therapy resistance seem to be inevitable. Therefore, much attention and effort have drifted from killing with agents to regulating with molecules of biology activity. miRNAs manipulation of stem cells’ renewal was reported to be effective, and several members of miRNAs could block ESR1 induction of oncogenic activation by binding to and degrading the 3′UTR of mRNAs.^7^, ^30^, ^31^, ^32^ In this study, we gave special attention to the comprehensive interactions among the non‐coding genes and revealed the uncovered relationship of lncRNA‐miRNAs in controlling the division manners and subsequent renewal ability.

We first identified the clinical indications of Let‐7 family and Wnt signalling pathway in patients with breast cancer; we used public big data to primarily establish the proposed interactions between Let‐7, H19 and Wnt signalling. Through using EdU green dye staining, we could identify the status of dividing cells, which was naturally symmetric division when it happened in cancer stem cells. Let‐7c inhibited the symmetric division and therefore decreased the forming numbers of spheres, which was similar to previous findings. H19 overexpression and Wnt pathway stimulated the symmetric division effectively, and Let‐7c counteracting the promoting roles of Wnt signalling, we therefore hypothesized the signalling cascade of H19/Let‐7c/ESR1/Wnt. Moreover, sh‐H19 and Wnt activity regulators of LiCl, Wnt3a and Axitinib were used to dig out the significance of their existence and defecting. In H19 dominated symmetric division, Let‐7c functions were antagonized by sponge molecular of H19, reactivating the ESR1/Wnt expression and activity, forming the straight‐forward pathway of H19/Let‐7c/Wnt. Interestingly, the unexpected discovery of Wnt signalling promoted H19 expression supplemented the one‐way cascade, helping to circle the H19 and Wnt in a H19/Let‐7c/Wnt regulatory loop, functioning in the renewal of BrCSCs.

Asymmetric cell division helps to constrain the stem cells pool, however, was often lost, in division of cancer stem cells. The symmetric dividing cells could expand the stem cells pool more effectively, generating the perpetual motion machine of cancer stem cells to maintain the tumour group. Many kinds of novel pharmaceutics have been tentatively applied in therapeutic experiments in vitro and in vivo, and the family of non‐coding genes were given high expectations for their crucial roles in participating in nearly all procedures of stem cells’ biology. However, manipulation of a non‐coding failed to conquer the stem cells renewal, as much more complicated network has connected these genes, especially the non‐coding genes into one complex. Our results also suggested that antagonizing one malignant lncRNA or enforcing one certain miRNA will disturb the established cascade, and the way of using clusters or groups of interrelated factors seems to be the only possible and sensible strategy.

## CONFLICT OF INTEREST

Authors declare each has approved this article to be published and that this research was conducted in the absence of any commercial or financial relationship that could be construed as a potential conflict of interest.
