# Supplementary figures and images for: H19 regulation of oestrogen induction of symmetric division is achieved by antagonizing Let‐7c in breast cancer stem‐like cells
Source: Cell Prolif. 2018 Oct 18;52(1):e12534. doi: 10.1111/cpr.12534 (PMC6430450; doi:10.1111/cpr.12534)

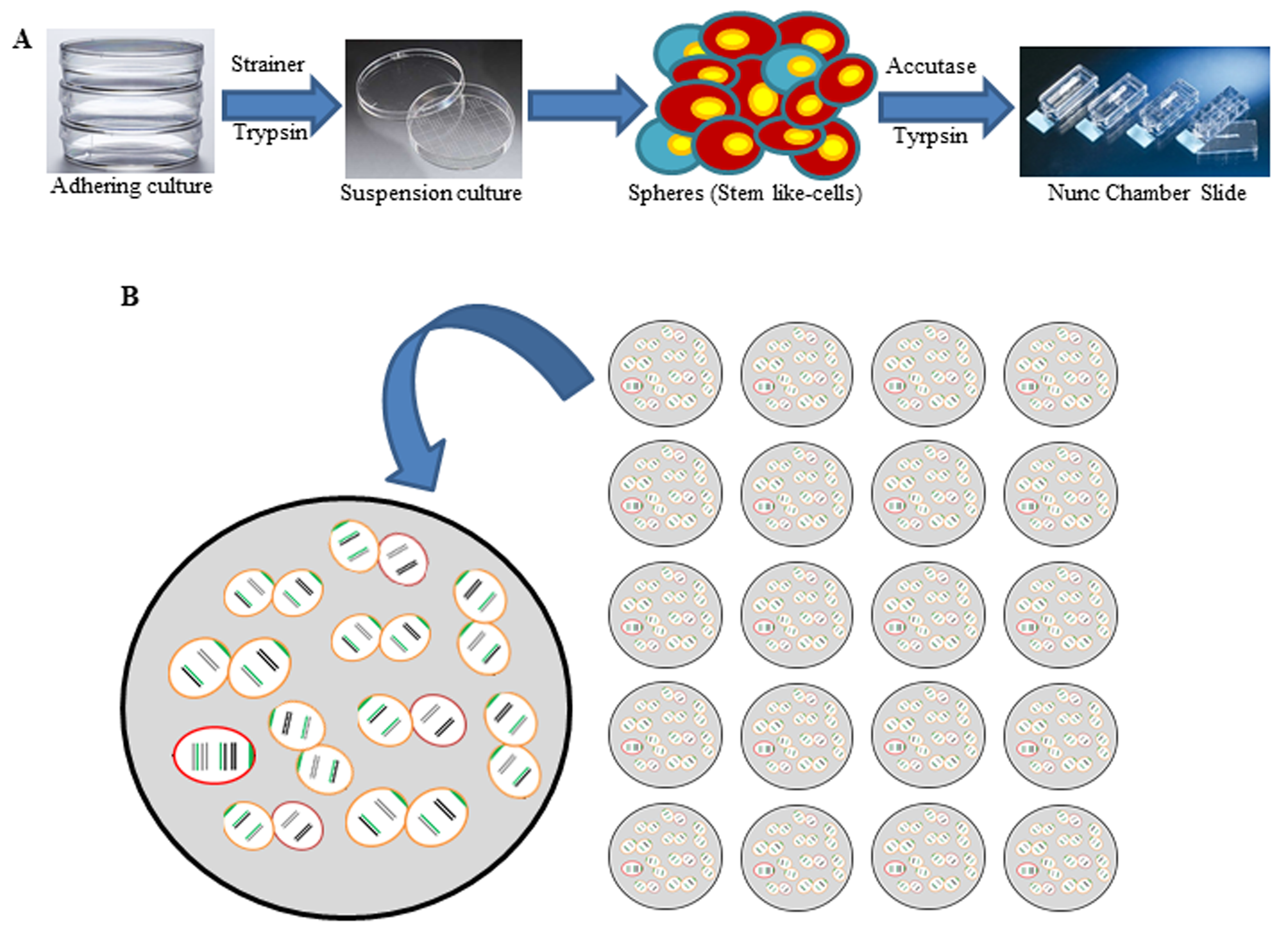

Supplement: Supplementary file 1 [file CPR-52-e12534-s001.tif]

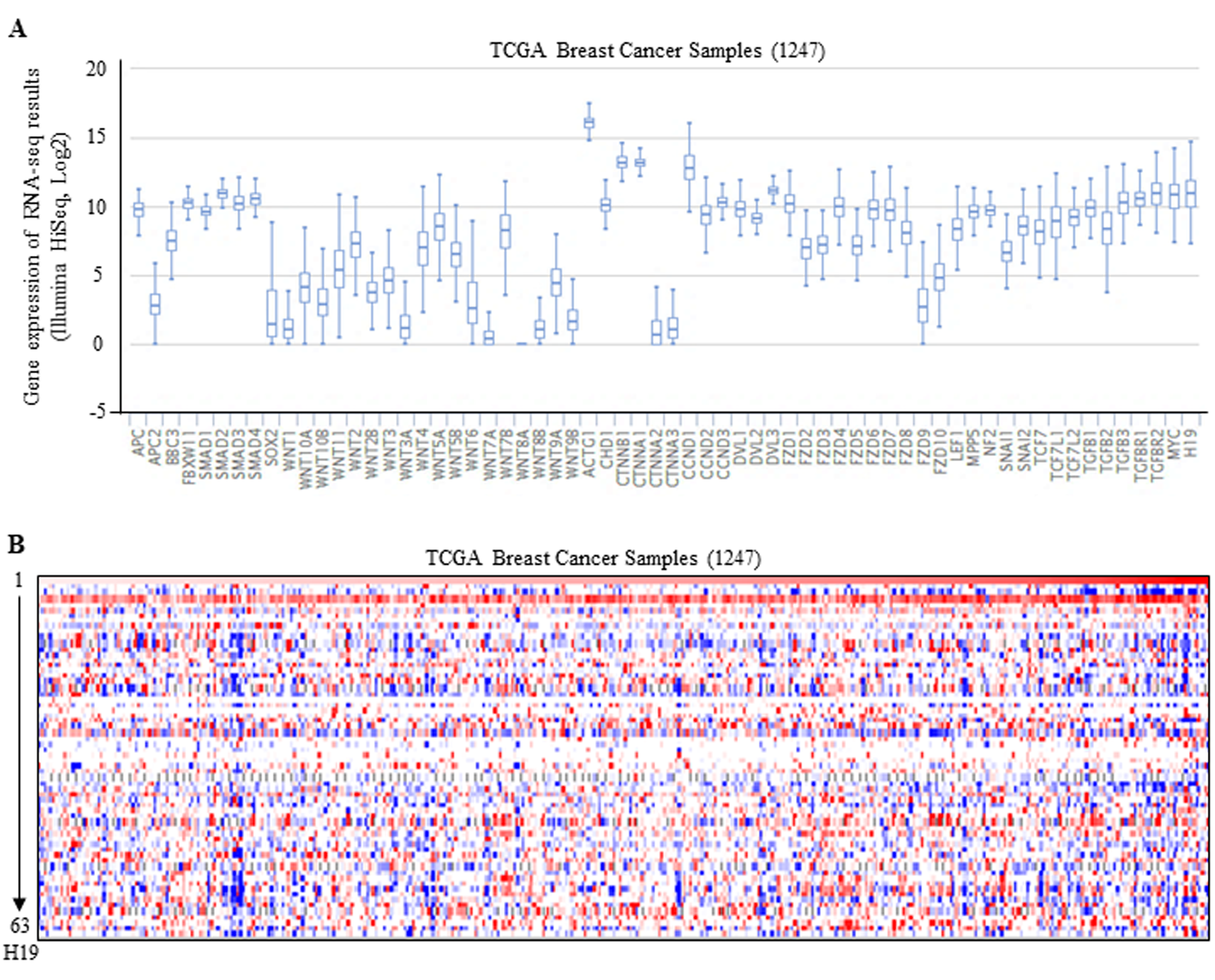

Supplement: Supplementary file 2 [file CPR-52-e12534-s002.tif]

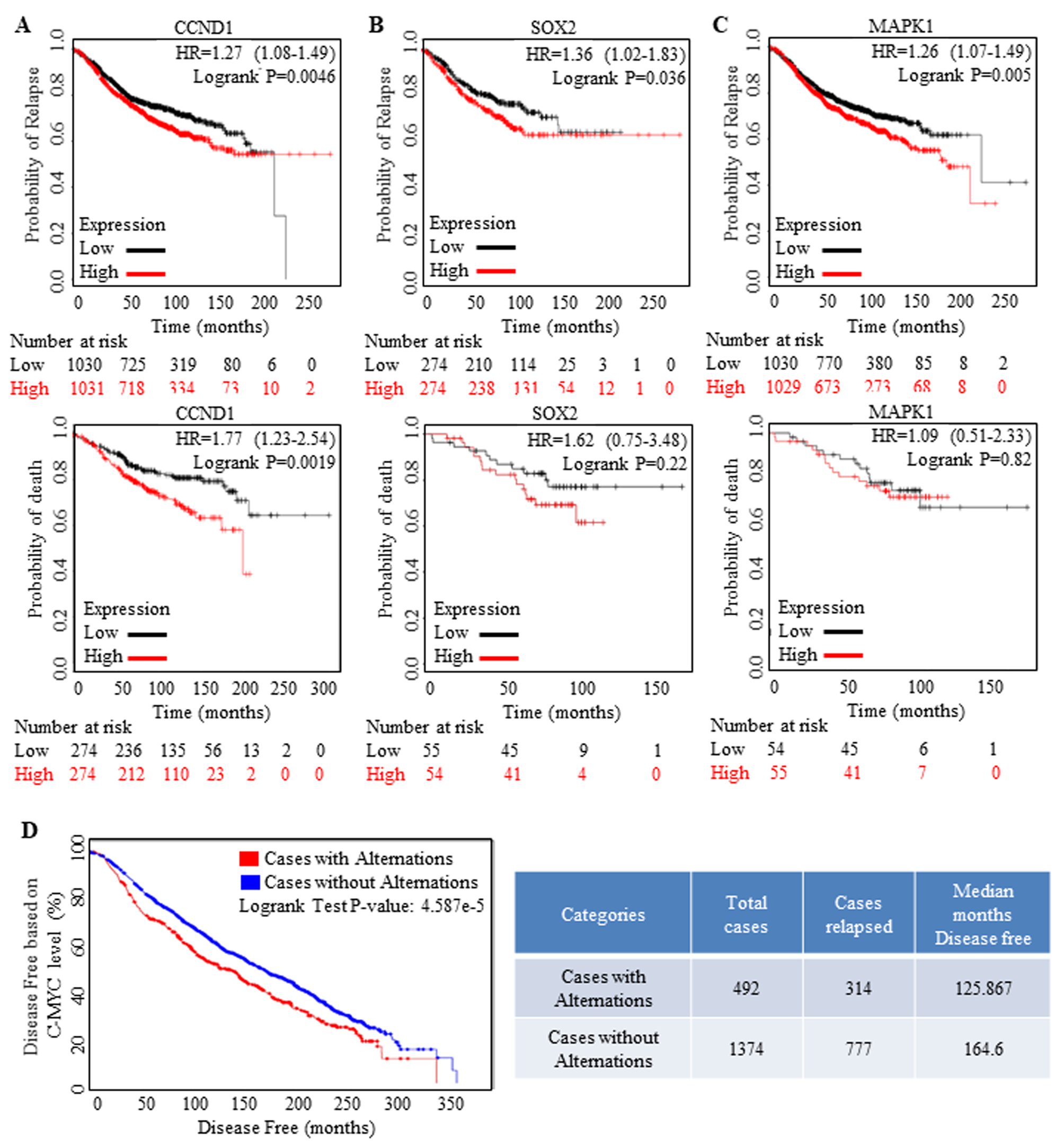

Supplement: Supplementary file 3 [file CPR-52-e12534-s003.tif]
