# Supplementary material for: H19 regulation of oestrogen induction of symmetric division is achieved by antagonizing Let‐7c in breast cancer stem‐like cells
Source: Cell Prolif. 2018 Oct 18;52(1):e12534. doi: 10.1111/cpr.12534 (PMC6430450; doi:10.1111/cpr.12534)
Supplement: Supplementary file 4 [file CPR-52-e12534-s004.docx]

Supplemental Figure Legends

Figure S1 Illustration of instructions referring spheres formation and dividing stem cells’ detection

***A*** Spheres were enriched for acquiring the stem-like cells, and then the single stem cell was seed in chamber slide after being filtrated. ***B*** Twenty images were taken when performing the division study, and the asymmetric and symmetric ratio were calculated as a mean number.

Figure S2 Correlation between Certain Wnt signaling factors and prognosis of patients with breast cancer

Main and crucial members of Wnt signaling were selected and applied for prognosis evaluation. ***A*** Increased CCND1 expression level indicated higher ratio of relapse probability (above), and the shorter survival time (below), with the differences being significant. ***B*** A. Increased SOX2 expression level indicated higher ratio of relapse probability (above), however, significant difference did not occur when comparing the survival time (below). ***C*** A. Increased MAPK1 expression level indicated higher ratio of relapse probability (above), the difference was not significant in groups harboring deregulated MAPK1 expression when analyzing the over survival time. ***D*** C-BIOPORTAL was applied for C-MYC function analysis, and in patients with increased C-MYC, disease free survival time was much shorter than that of control group implying lower C-MYC level.

Figure S3 TCGA study of Wnt activation and deregulated H19 in breast cancer tissues

***A*** RNA sequence results of 1247 patients were used to conduct the clinical analysis, and Wnt signaling factors, together with H19, were usually activated and increased in patients with breast cancer. ***B*** The full results of heat map information referring to Wnt signaling, and genes of “1.APC 2.APC2 3.BBC3 4.FBXW11 5.SMAD1 6.SMAD2 7.SMAD3 8.SMAD4 9.SOX2 10.WNT1 11.WNT10A 12.WNT10B 13.WNT11 14.WNT2 15.WNT2B 16.WNT3 17.WNT3A 18.WNT4 19.WNT5A 20.WNT5B 21.WNT6 22.WNT7A 23.WNT7B 24.WNT8A 25.WNT8B 26.WNT9A 27.WNT9B 28.ACTG1 29.CHD1 30.CTNNB1 31.CTNNA1 32.CTNNA2 33.CTNNA3 34.CCND1 35.CCND2 36.CCND3 37.DVL1 38.DVL2 39.DVL3 40.FZD1 41.FZD2 42.FZD3 43.FZD4 44.FZD5 45.FZD6 46.FZD7 47.FZD8 48.FZD9 49.FZD10 50.LEF1 51.MPP5 52.NF2 53.SNAI1 54.SNAI2 55.TCF7 56.TCF7L1 57.TCF7L2 58.TGFB1 59.TGFB2 60.TGFB3 61.TGFBR1 62.TGFBR2 63.MYC” were arranged in order from the top down, with H19 being at the bottom. This map result was displayed as supplement of Figure 2.
